# NHHR: An Important Independent Risk Factor for Patients with STEMI

**DOI:** 10.31083/j.rcm2312398

**Published:** 2022-12-09

**Authors:** Po Gao, Jing Zhang, Xizhen Fan

**Affiliations:** ^1^Department of Cardiovascular Medicine, Hefei Second People's Hospital, Hefei Hospital Affiliated to Anhui Medical University, 230000 Hefei, Anhui, China; ^2^Department of Cardiovascular Medicine, The First Affiliated Hospital of University of Science and Technology of China, Anhui Provincial Hospital, 230000 Hefei, Anhui, China

**Keywords:** STEMI, NHHR, LDL-C, non-HDL-C, LDL-C/HDL-C, Gensini score

## Abstract

**Background:**

In this study, we investigated whether the ratio of non-high-density lipoprotein cholesterol to high-density lipoprotein cholesterol (NHHR) is associated with the development of acute ST-segment elevation myocardial infarction (STEMI).

**Methods:**

889 STEMI patients who had not previously received lipid-lowering therapy were selected as the test group and 120 patients with less than 50% coronary stenosis were selected as the control group. All patients completed the related blood tests the morning after admission, and Gensini scores were based on coronary angiography results. The differences were compared using a *t*-test, rank sum test, chi-square test and logistic regression analysis. Linear regression analysis was used to study the correlation between variables. Receiver Operating Characteristic (ROC) curves were used to validate the predictive value of NHHR for STEMI.

**Results:**

NHHR was shown to be a significant independent risk factor for STEMI according to binary logistic regression analysis (OR = 0.163, 95% CI: 0.065–0.411, *p *< 0.05). There were shown to be differences in the NHHR depending on the gender of the STEMI patients (z = –1.663, *p *< 0.1). Linear regression analysis revealed a stronger correlation between NHHR and Gensini score (r = 0.394, *p *< 0.05) in the test group. Finally, we demonstrated that NHHR has a good predictive effect on STEMI, using an ROC curve (Area Under Curve (AUC): 0.818, 95% CI: 0.777–0.859, *p *< 0.05).

**Conclusions:**

NHHR is a good predictor of coronary artery disease severity in STEMI patients and an important independent risk factor for STEMI, especially for patients who have not received lipid-lowering treatment in the past, and male STEMI patients need more stringent lipids management than female STEMI patients.

## 1. Introduction

Cardiovascular diseases (CVD) account for one-third of all deaths in the world, and two-thirds of them occur in developing countries. In recent years, CVD has accounted for more than 40% of all deaths among Chinese residents, about 45% in urban areas, and about 42% in rural areas, which is a significantly higher percentage than that of other diseases such as cancer. It has become the leading cause of death among Chinese residents and is still showing an upward trend [1]. Previous studies have recognized that low-density lipoprotein cholesterol (LDL-C) is significantly correlated with atherosclerosis, so LDL-C has always been the primary target of lipid-lowering therapy. However, although the LDL-C level of patients has been effectively controlled, the prevalence of atherosclerotic cardiovascular disease (ASCVD) is still high. Subsequent studies have begun to focus on non-high-density lipoprotein cholesterol (non-HDL-C). Some studies have found that compared with LDL-C, non-HDL-C has a stronger correlation with cardiovascular events and atherosclerosis and is a better predictor and prevention indicator [2, 3]. In 2016, the expert consensus committee of the American College of Cardiology (ACC) proposed that the non-HDL-C index can be used the same as the LDL-C index in patients with diabetes and elevated triglycerides. In 2017, the ACC expert consensus committee issued the latest guidelines on the role of non-statins in the prevention of coronary artery disease [4]. This is an updated version of the ACC guidelines from 2016, which considers non-HDL-C to be a target for all risk groups. In recent years, the ratio of non-high-density lipoprotein cholesterol to high-density lipoprotein cholesterol (NHHR) has attracted increasing attention, and many studies have proposed that it can reflect the relationship between mixed blood lipids, better reflect the balance between atherosclerosis and anti-atherosclerosis, and better reflect dyslipidemia than the traditional blood lipid spectrum. NHHR has been found to be significantly associated with a variety of lipid abnormality-related diseases such as atherosclerotic plaques [5], metabolic syndrome, insulin resistance [6, 7], nonalcoholic fatty liver [8], and chronic kidney disease [9]. Numerous studies have shown that NHHR can better assess the severity of coronary artery disease and cardiovascular adverse events than LDL-C, HDL-C, non-HDL-C, LDL-C/HDL-C, and other single indicators [10, 11]. This study focuses on STEMI, the most severe form of coronary heart disease, and provides additional guidance for lipids management in STEMI patients.

## 2. Methods

**Fig. 1.**
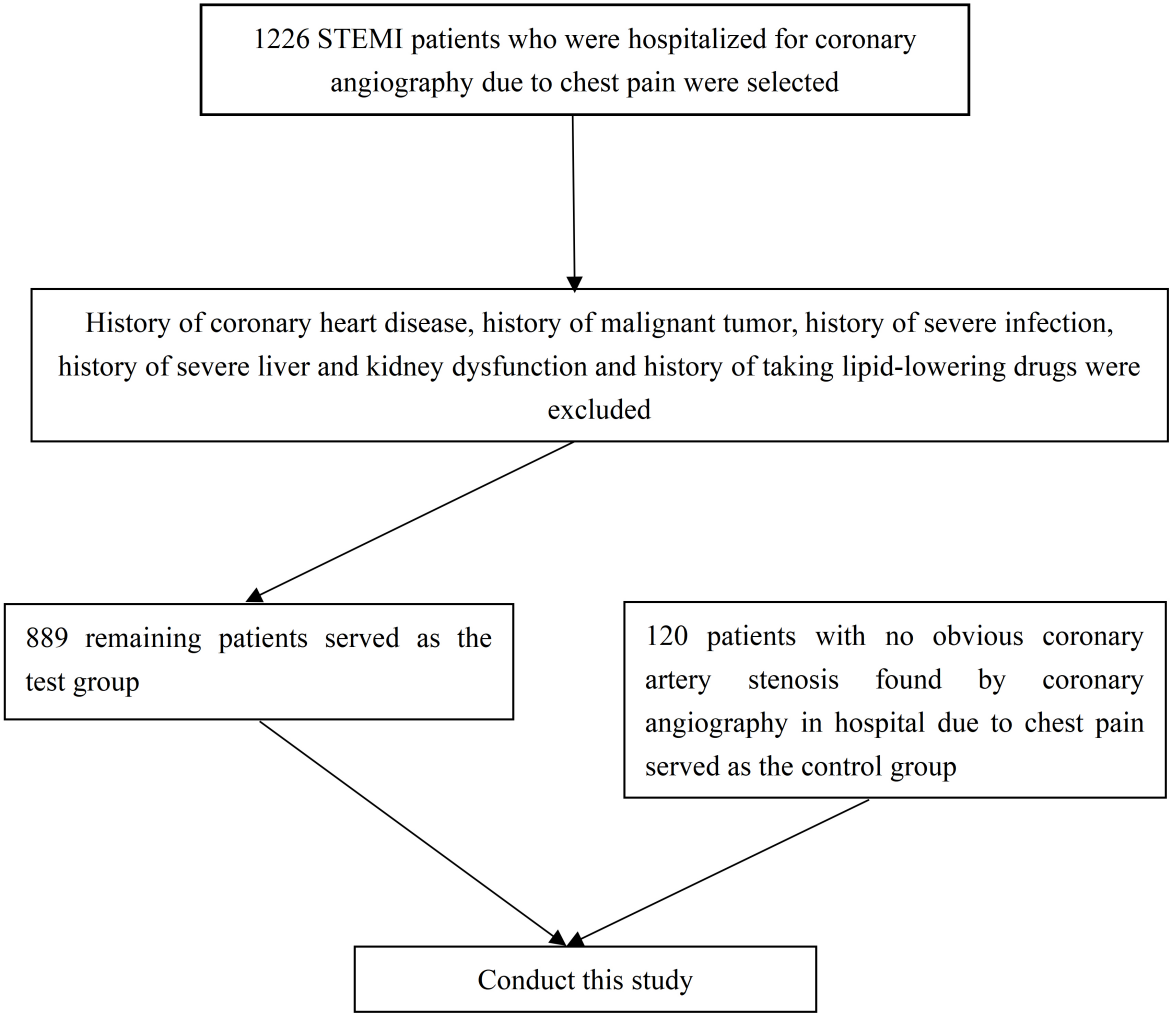
**Screening of patients in this study**.

1226 STEMI patients with chest pain who had been hospitalized for coronary angiography were selected. All patients with a history of coronary heart disease, malignant tumor, severe infection, severe liver and kidney dysfunction, or of taking lipid-lowering drugs were excluded. 889 remaining STEMI patients served as the test group and 120 patients with less than 50% coronary stenosis were selected as the control group (Fig. 1). The diagnostic criteria refer to the “Global Definition of the Fourth Myocardial Infarction” promulgated in 2018 [12]. The sex, age, history of diabetes, history of hypertension, smoking habits, height, and weight of each patient was recorded. All of the selected patients underwent blood tests, electrocardiograms, and coronary angiography upon admission. A full lipid profile blood test was performed the next morning. Two doctors detailed the location and stenosis of each patient’s coronary artery disease. The degree of coronary artery disease was measured using the Gensini score. Whenever there was any disagreement, a third physician would join the discussion in order to help come to a decision. Non-HDL-C equals total cholesterol (TC) minus HDL-C, and NHHR equals non-HDL-C divided by HDL-C.

## 3. Statistical Analysis

All data were analyzed and processed using IBM SPSS Statistics version 26 (IBM SPSS Inc., Chicago, IL, USA). Non-normal distribution data between the two groups were expressed by rank sum test, results by M (Q1, Q3), counting data by Chi-square test, and results by χ^2^. Correlation between variables was analyzed using Pearson linear correlation analysis or logistic regression analysis, and the results were expressed as correlation coefficient r or ratio or 95% confidence interval (95% CI), and *p *< 0.1 was considered significant for all analytical methods.

## 4. Results

### 4.1 Clinical Data Characteristics of All Patients

The patients were divided into a test group (STEMI group) and a control group (less than 50% coronary stenosis) based on coronary angiography results. There were 889 patients in the test group, of which 676 were male and 213 were female, and there were 120 patients in the control group. Table 1 summarizes the clinical data of all patients.

**Table 1.** **Clinical data characteristics of all patients**.

Variables	Test group (n = 889)	Control group (n = 120)	χ^2^/z	*p*
age	62.71 (53.00, 72.00)	58.98 (50.00, 69.00)	–2.745	0.006
Sex (%)	Male 676 (76%)	Male 80 (67%)	4.940	0.026
	Female 213 (24%)	Female 40 (33%)		
BMI (${\mathrm{Kg/m}}^{2}$)	23.72 (22.00, 26.00)	22.52 (20.00, 24.00)	–4.163	0.000
Hypertension (%)	512 (58%)	55 (46%)	5.934	0.015
Diabetes (%)	367 (43%)	33 (28%)	8.438	0.004
Smoking (%)	516 (58%)	43 (36%)	21.086	0.000
ANC (×${\mathrm{10}}^{9}$)	6.20 (4.59, 7.00)	4.00 (3.26, 4.50)	–11.587	0.000
TLC (×${\mathrm{10}}^{9}$)	1.88 (1.48, 2.26)	2.12 (1.53, 2.64)	–3.969	0.000
MONOC (×${\mathrm{10}}^{9}$)	0.60 (0.42, 0.73)	0.43 (0.31, 0.50)	–7.899	0.000
RBC (×${\mathrm{10}}^{9}$)	4.38 (3.96, 4.84)	4.38 (3.93, 4.78)	–0.026	0.979
RDW	13.21 (12.50, 13.50)	13.18 (12.50, 13.50)	–0.196	0.845
TC (mmol/L)	4.42 (3.73, 4.98)	3.69 (3.09, 4.37)	–7.621	0.000
TG (mmol/L)	1.66 (1.04, 1.89)	1.39 (0.92, 1.66)	–2.897	0.004
HDL-C (mmol/L)	0.93 (0.78, 1.04)	1.12 (0.92, 1.23)	–7.262	0.000
LDL-C (mmol/L)	2.66 (20.7, 3.18)	1.86 (1.49, 2.30)	–10.342	0.000
VLDL-C (mmol/L)	0.70 (0.44, 0.85)	0.61 (0.38, 0.75)	–2.843	0.017
Non-HDL-C (mmol/L)	3.49 (2.81, 4.03)	2.56 (2.00, 3.14)	–9.803	0.000
LDL-C/HDL-C	2.99 (2.27, 3.51)	1.73 (1.29, 2.14)	–8.68	0.000
NHHR	3.97 (2.96, 4.69)	2.43 (1.69, 3.09)	–11.328	0.000
Gensini score	71.32 (50.00, 88.00)	19.46 (16.00, 30.00)	–17.629	0.000

Remarks: ANC, Absolute Neutrophil Count; TLC, Total Lymphocyte Count; MONOC, Monocyte Count; RBC, Red Blood Cell Count; RDW, Red blood cell distribution width; TG, triglyceride; BMI, Body Mass Index.

It is evident that there are significant differences in blood lipid indicators between the two groups.

### 4.2 Correlation between Various Indices in Blood Lipid Test and Gensini Score

In the test group, we found that TC, TG, HDL-C, LDL-C, non-HDL-C, LDL-C/HDL-C and NHHR were significantly correlated with Gensini score (Table 2).

**Table 2.** **Correlation between various indices in blood lipid test and Gensini score**.

Variables	r	*p*
TC (mmol/L)	0.318	0.000
TG (mmol/L)	0.068	0.042
HDL-C (mmol/L)	–0.171	0.000
LDL-C (mmol/L)	0.403	0.000
VLDL-C (mmol/L)	0.004	0.902
Non-HDL-C (mmol/L)	0.361	0.000
LDL-C/HDL-C	0.475	0.000
NHHR	0.394	0.000

### 4.3 Multivariate Linear Regression Analysis of Serum Lipid Indices and Gensini Scores

Age, sex, hypertension, diabetes, and smoking are all important risk factors for coronary heart disease. We used the Gensini scores of the test group as a factor and used all of the relevant indicators, as well as age, sex, smoking, hypertension, and diabetes as independent variables for a step-by-step multivariate linear regression analysis. Although LDL-C/HDL-C was found to have a stronger correlation with Gensini score (Table 3b Model 2), NHHR was also shown to have a significant correlation with Gensini score (Table 3a Model 1) ($R^{2}$ = 0.246, β = 0.431, 95% CI: 5.694–9.012, *p *< 0.05). A higher NHHR was associated with more severe coronary artery disease in STEMI patients.

**Table 3a.** **Multivariate linear regression analysis of serum lipid indices and Gensini scores**.

Variables	SD	β	t	95% CI	*p*
Age	0.058	0.075	2.554	0.035, 0.264	0.011
Hypertension	1.546	0.089	2.999	1.602, 7.669	0.003
Diabetes	1.549	0.067	2.284	0.498, 6.576	0.023
LDL-C (mmol/L)	1.425	0.412	9.071	10.127, 15.719	0.000
Non-HDL-C (mmol/L)	1.713	–0.285	–4.404	–10.906, –4.182	0.000
NHHR	0.845	0.431	8.700	5.694, 9.012	0.000

**Model 1**: Research factors included (age, sex, hypertension, diabetes, smoking, TC, TG, HDL-C, LDL-C, non-HLD-C, NHHR).

**Table 3b.** **Multivariate linear regression analysis of serum lipid indices and Gensini scores**.

Variables	SD	β	t	95% CI	*p*
Age	0.059	0.076	2.574	0.036, 0.266	0.010
Hypertension	1.547	0.090	3.025	1.643, 7.717	0.003
Diabetes	1.539	0.069	2.338	0.578, 6.618	0.020
TC (mmol/L)	0.914	0.077	2.205	0.221, 3.808	0.028
LDL-C/HDL-C	0.801	0.433	12.335	8.307, 11.450	0.000

**Model 2**: Research factors included (age, sex, hypertension, diabetes, smoking, TC, TG, HDL-C, LDL-C, non-HLD-C, LDL-C/HDL-C, NHHR).

### 4.4 NHHR is an Important Independent Risk Factor in STEMI Patients

We performed a multi-factor binary logistic regression analysis on age, sex, hypertension, diabetes, smoking, TC, TG, LDL-C, HDL-C, non-HDL-C, LDL-C/HDL-C, and NHHR in the test group and the control group. NHHR was found to be an important independent risk factor for STEMI (OR = 0.163, 95% CI: 0.065–0.411, *p *< 0.05). This further indicates that NHHR is a better index predictor of STEMI (Table 4).

**Table 4.** **Binary multivariate logistic regression analysis**.

Variables	β	SE	Wald	OR	95% CI	*p*
Sex	–0.740	0.297	6.198	0.477	0.477, 0.267	0.013
Age	–0.039	0.010	14.964	0.961	0.942, 0.981	0.000
Hypertension	–0.901	0.253	12.720	0.406	0.248, 0.666	0.000
Diabetes	–0.204	0.271	0.783	0.787	0.463, 1.338	0.376
Smoking	–0.450	0.278	2.627	0.638	0.370, 1.099	0.105
TC (mmol/L)	2.221	1.008	4.854	9.217	1.278, 66.468	0.028
TG (mmol/L)	–0.124	0.164	0.566	0.884	0.641, 1.219	0.452
HDL-C (mmol/L)	–1.838	1.473	1.556	0.159	0.009, 2.856	0.212
LDL-C (mmol/L)	–3.603	1.702	4.481	0.027	0.001, 0.766	0.034
LDL-C/HDL-C	0.797	1.639	0.237	2.219	0.089, 55.102	0.627
NHHR	–2.182	0.907	5.792	0.113	0.019, 0.667	0.016

### 4.5 LDL-C, non-HDL-C, and NHHR Predict STEMI

**Fig. 2.**
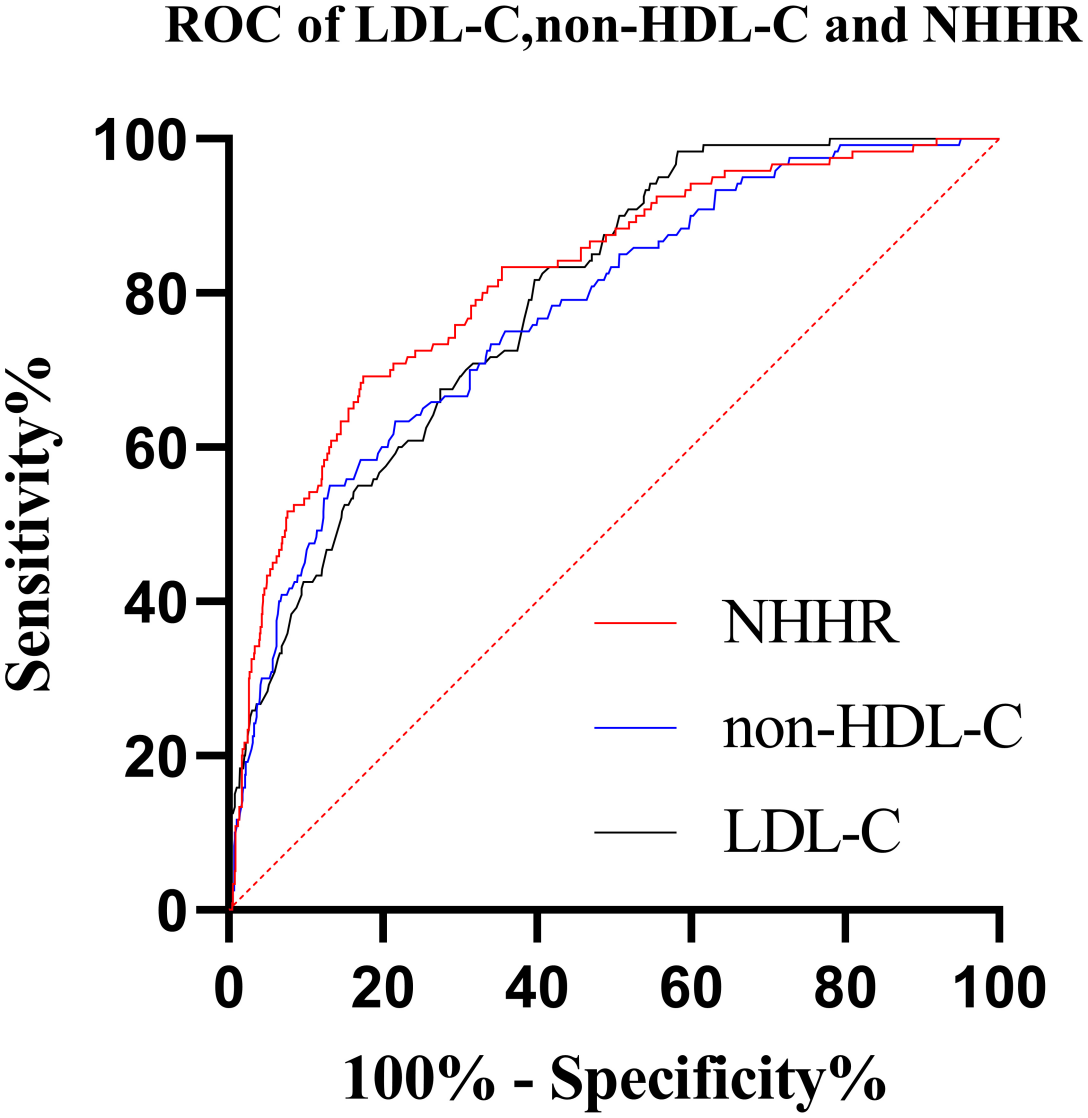
**ROC curves of patients’ LDL-C, non-HDL-C, and NHHR**.

LDL-C, non-HDL-C, and NHHR were used as ROC curves between the test and control groups (Table 5, Fig. 2). NHHR better predicts whether patients with chest pain have STEMI.

**Table 5.** **LDL-C, non-HDL-C, and NHHR Predict STEMI**.

Variables	AUC	Cutoff value	Sensitivity	Specificity	95% CI	*p*
LDL-C (mmol/L)	0.790	2.35	0.603	0.817	0.752, 0.829	0.000
Non-HDL-C (mmol/L)	0.776	2.52	0.868	0.550	0.731, 0.820	0.000
NHHR	0.818	2.68	0.826	0.692	0.777, 0.859	0.000

### 4.6 Differences in Blood Lipids in Each Gender of STEMI Patients

With the deepening of the study, we also found differences in blood lipids in the different genders of STEMI patients. We divided all STEMI patients into two groups according to gender and compared the differences in blood lipids between the two groups. We found that the blood lipid level in male STEMI patients was lower than in female patients, and that the difference was statistically significant (Table 6). This indicates that male STEMI patients need more strict lipid management.

**Table 6.** **Blood lipid differences in STEMI patients of different genders**.

Variables	Male group (n = 676)	Female group (n = 213)	z	*p*
TC (mmol/L)	4.40 (3.72, 4.98)	4.49 (3.86, 5.01)	–0.839	0.401
TG (mmol/L)	1.66 (1.02, 1.88)	1.66 (1.07, 1.93)	–1.118	0.264
HDL-C (mmol/L)	0.93 (0.78, 1.03)	0.94 (0.78, 1.08)	–0.741	0.459
LDL-C (mmol/L)	2.65 (2.06, 3.16)	2.70 (2.11, 3.22)	–0.963	0.336
Non-HDL-C (mmol/L)	3.47 (2.80, 4.02)	3.55 (2.82, 4.13)	–1.233	0.218
LDL-C/HDL-C	2.98 (2.27, 3.50)	3.01 (2.27, 3.55)	–0.137	0.891
NHHR	3.95 (2.94, 4.68)	4.04 (3.02, 4.74)	–1.663	0.096

**Fig. 3.**
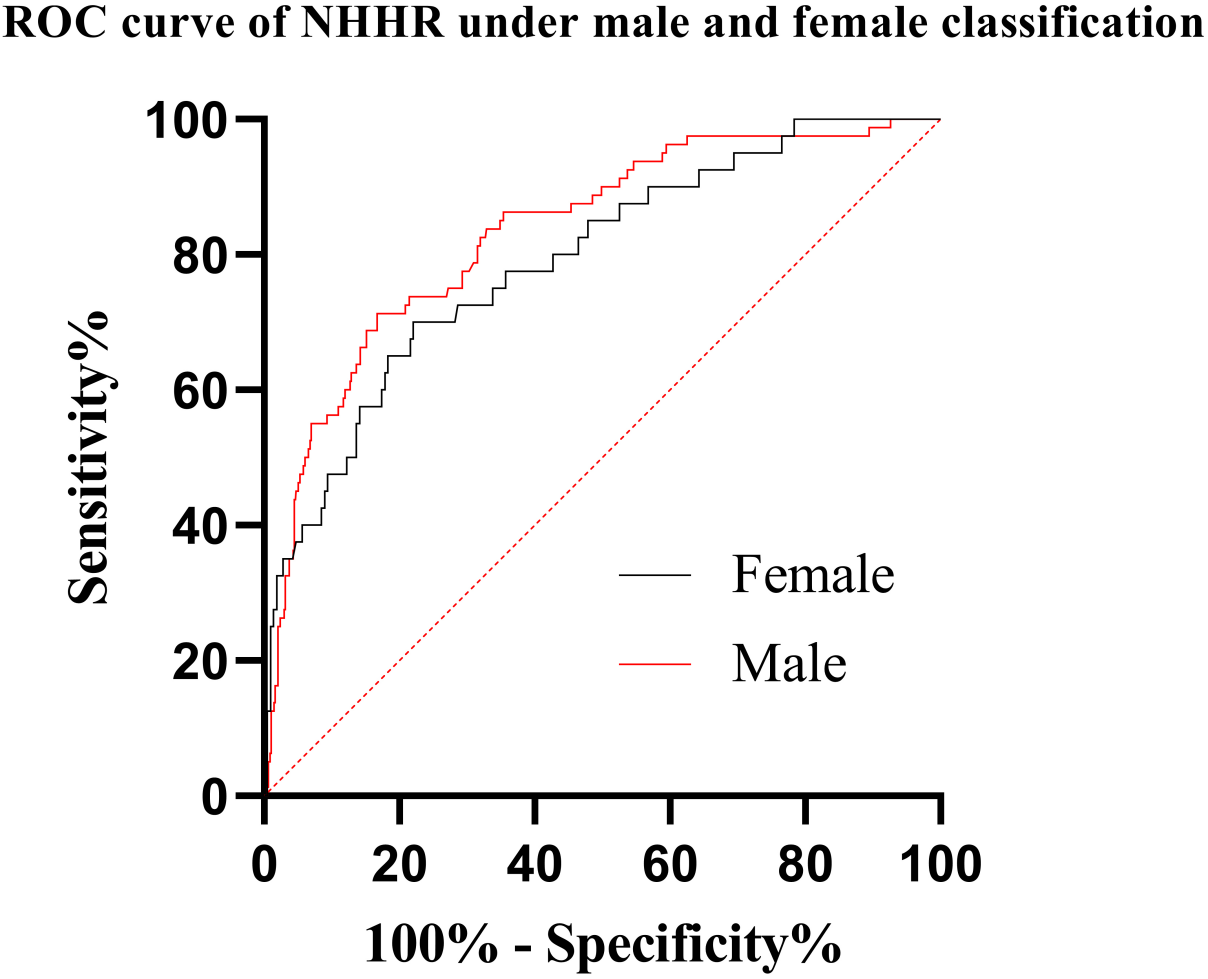
**ROC curve of NHHR under male and female classification**.

We divided 1009 patients into a male group and a female group for a separate study, and each group (male and female) was divided into a test group and a control group. Male patients with STEMI served as the male test group 1 (n = 676) and male patients with less than 50% coronary stenosis served as the male control group 1 (n = 80). Female patients with STEMI served as the female test group 2 (n = 213), and female patients with less than 50% coronary stenosis served as the female control group 2 (n = 40). Using NHHR as an independent variable, the ROC curves of male and female classification were drawn respectively (Fig. 2). In the male category, The ROC curve of the NHHR was (AUC: 0.833, 95% CI: 0.785–0.881; *p *< 0.05). When the cutoff value is 2.66, the sensitivity is 0.833 and the specificity is 0.712. In the female category, The ROC curve of the NHHR was (AUC: 0.791, 95% CI: 0.713–0.889; *p *< 0.05). When the cutoff value is 2.94, the sensitivity is 0.779 and the specificity is 0.700. This further indicates that male STEMI patients need more strict lipid management (Fig. 3).

## 5. Discussion

It is evident that lipid abnormality is an important risk factor for coronary heart disease (CHD). LDL-C is an important risk factor for CHD and can also serve as an important index to evaluate the management of blood lipids in CHD.

Even if the LDL-C of the patient drops below 1.4 mmol/L, as is recommended by the guidelines, the patient is still likely to suffer from coronary heart disease. This is called residual risk. Clinicians are still looking for better indicators to manage the residual risk of CHD [13]. Therefore LDL-C/HDL-C, non-HDL-C, and the NHHR are factors of concern. Changes in these ratios are proven indicators for assessing the risk of coronary heart disease [14, 15]. Non-HDL-C refers to cholesterol carried by apolipoprotein B (apo B) particles that promote atherosclerosis, reflecting the content of TG-rich lipoproteins including LDL C, medium-density lipoprotein, very low-density lipoprotein cholesterol (VLDL-C), and its byproducts. TG-rich lipoproteins participate in the occurrence and development of atherosclerotic lesions through a variety of mechanisms. One of these mechanisms is the entering of the arterial endothelium, where they are phagocytized by macrophages to form foam cells, thus triggering the occurrence and development of atherosclerotic lesions. All these lipoproteins can bring cholesterol into the arterial wall and lead to atherosclerotic lesions [3]. The guidelines also suggest that attention should be paid to non-HDL-C, especially in patients with diabetes, obesity, or metabolic syndrome, in whom the symptoms are often manifested as non-HDL-C, TG level increase, and HDL-C level decrease, while their LDL-C level may not be high [4]. In addition, the calculation method of non-HDL-C is simple. HDL-C is subtracted from TC, and it is not affected by fasting conditions, which is convenient for patients. Also, it is not affected by TG variation, which makes it highly reliable. The newly published 2019 European Society of Cardiology/European Society of Atherosclerosis (ESAC/EAS) guidelines further adjusted their recommendations regarding LDL-C and non-LDL-C. For patients with extremely high cardiovascular risk, the recommended primary target is LDL-C <1.4 mmol/L (or >50% reduction in LDL-C), and the recommended secondary target is non-HDL-C <2.2 mmol/L [13].

At present, there have been many studies on NHHR. Studies have proven that NHHR can effectively predict diabetes [16]. Compared with non-HDL-C, NHHR can more accurately represent the balance between proatherogenic lipoproteins and antiatherogenic lipoproteins, indicating a more comprehensive lipid dysregulation. Recent studies have found that TC and LDL-C levels in patients with acute coronary syndrome decrease with age [17]. These results suggests that it may be unreasonable for people of different ages to define their degree of dyslipidemia by referring to the same LDL-C or non-HDL-C standard reference range, and it may not be an accurate way to evaluate the occurrence, severity, or prognosis of coronary heart disease. In comparison, the lipid ratio, especially the NHHR, may provide a more objective basis for evaluation. Previous studies have mostly focused on the broad field of coronary heart disease. Our study focused specifically on STEMI, the most severe form of coronary heart disease. We found a positive correlation between NHHR and the severity of coronary artery disease in STEMI patients (r = 0.394, *p *< 0.05). The higher the NHHR, the more severe the coronary artery disease. Compared with LDL-C and non-HDL-C, NHHR was a better predictor of STEMI in patients with chest pain (AUC: 0.818, 95% CI: 0.777–0.859, *p *< 0.05). Not only is NHHR an independent risk factor for STEMI (OR = 0.163, 95% CI: 0.065–0.411, *p *< 0.05), but it is also significant in that there are differences in NHHR between genders of STEMI patients, and men may require more rigorous lipid management. This may be related to hormone production and poor living habits such as smoking in male patients. The effects of estrogen on STEMI include the following [18, 19, 20]: ① It can affect lipoprotein and lipid metabolism, accelerate the clearance of chyle particles in the liver, increase the secretion of very low-density lipoprotein cholesterol (VLDL-C) in the liver, up-regulate LDL-C receptor sites in the liver, accelerate LDL-C uptake in the liver, promote apolipoprotein A (apo A) synthesis, accelerate HDL-C metabolism, accelerate the secretion of cholic acid, promote the elimination of cholesterol from the body, thereby reducing LDL-C and TC, and increase HDL-C. ② It can provide a sustained, low level of antioxidant activity, slowly and persistently reduce the production of oxidized low-density lipoprotein cholesterol (ox-LDL-C) under the intima, and have a protective effect on endothelial cells against damage by ox-LDL-C. ③ It has a protective effect against vascular injury. Some studies have shown that estrogen can promote the growth of endothelial cells. After vascular injury, estrogen can induce the increase of endothelial growth factor and promote the healing of endothelium, and can also inhibit the apoptosis of human endothelial cells through estrogen receptor-dependent processes. In addition, estrogen can inhibit the proliferation of vascular smooth muscle cells, promote the growth of endothelial cells, and play a long-term protective role in maintaining normal vascular function.

### Study Limitations

This is a single-center retrospective study with small sample size and few indicators. A lack of follow-up resulted in high patient loss rates and a lack of sufficient longitudinal study. There have been no further studies on the differences in NHHR in STEMI patients of different genders.

## 6. Conclusions

We found that NHHR was positively correlated with the severity of coronary lesions in STEMI patients, and was also an independent risk factor for STEMI, especially for patients who have not received lipid-lowering treatment in the past. We also found that the NHHR was different in STEMI patients of different genders, and male STEMI patients need more strict lipid management. We need more research to prove whether it is feasible to use NHHR to guide STEMI patients to manage blood lipids.

## Data Availability

The data used to support the findings of this study are available from the corresponding authors upon request.
